# Investigating Laser Ablation Process Parameters for the Fabrication of Customized Microneedle Arrays for Therapeutic Applications

**DOI:** 10.3390/pharmaceutics16070885

**Published:** 2024-06-30

**Authors:** Faisal Khaled Aldawood, Abhay Andar, Salil Desai

**Affiliations:** 1 Department of Mechanical Engineering, College of Engineering, University of Bisha, P.O. Box 001, Bisha 67714, Saudi Arabia; faldawood@ub.edu.sa; 2 Translational Life Science Technology, University of Maryland Baltimore County, 1000 Hilltop Circle, Baltimore, MD 21250, USA; aandar@umbc.edu; 3 Center for Excellence in Product Design and Advanced Manufacturing, North Carolina A&T State University, Greensboro, NC 27411, USA

**Keywords:** drug delivery, laser ablation, microneedle, transdermal, ytterbium laser

## Abstract

Microneedles are an innovation in the field of medicine that have the potential to revolutionize drug delivery, diagnostics, and cosmetic treatments. This innovation provides a minimally invasive means to deliver drugs, vaccines, and other therapeutic substances into the skin. This research investigates the design and manufacture of customized microneedle arrays using laser ablation. Laser ablation was performed using an ytterbium laser on a polymethyl methacrylate (PMMA) substrate to create a mold for casting polydimethylsiloxane (PDMS) microneedles. An experimental design was conducted to evaluate the effect of process parameters including laser pulse power, pulse width, pulse repetition, interval between pulses, and laser profile on the desired geometry of the microneedles. The analysis of variance (ANOVA) model showed that lasing interval, laser power, and pulse width had the highest influence on the output metrics (diameter and height) of the microneedle. The microneedle dimensions showed an increase with higher pulse width and vice versa with an increase in pulse interval. A response surface model indicated that the laser pulse width and interval (independent variables) significantly affect the response diameter and height (dependent variable). A predictive model was generated to predict the microneedle topology and aspect ratio varying from 0.8 to 1.5 based on the variation in critical input process parameters. This research lays the foundation for the design and fabrication of customized microneedles based on variations in specific input parameters for therapeutic applications in dermal sensors, drug delivery, and vaccine delivery.

## 1. Introduction

Microneedles provide a minimally invasive technology for delivering drugs via the skin for different therapeutic applications and have shown great promise in recent years. Microneedles have been expanded and employed in a range of applications, including drug delivery, vaccine delivery, cosmetics, and disease diagnostics [1,2,3,4]. Due to the benefits and relevance of using this technology to overcome the complications of certain drugs to penetrate the stratum corneum, microneedles have gained popularity in recent years. Microneedles provide a painless experience to eliminate the pain and discomfort associated with IV injections [5,6,7]. For that reason, this technology could be an optimal choice for people with trypanophobia and young patients [8]. Furthermore, the convenience of using microneedles for transdermal drug delivery applications does not necessitate the need for a trained individual [9,10,11,12]. Other advantages of using microneedles are that they reduce the risk associated with transmitting infection into the body and tissue damage [13,14,15,16,17,18]. In addition, microneedles have shown the capability of transporting a drug into the body’s circulation [19], monitoring health, and detecting and extracting information from bodies [20].

Different manufacturing methods for fabricating microneedles were proposed, such as lithography [21], injection molding [22], and additive manufacturing [23,24,25]. Kochhar et al. fabricated a microneedle array using a lithography approach, which successfully penetrated cadaver pig skin [26]. The study proposed a simple method (single-step process) considering polymerization time, UV light intensity, and the distance from the light as parameters that impacted and controlled the length and tip diameter of microneedles. Li et al. used injection molding to fabricate hollowed titanium porous microneedle arrays [27]. The study performed several insertion tests on the human forearm and rabbit skin, concluding that microneedles successfully penetrated the skin without fracture. With the recent progress of additive manufacturing technology, several studies have used this technology to enhance the fabrication of microneedles [28,29]. Johnson et al. were the first to fabricate microneedle master molds using commercial 3D printing [30]. With the manipulation of parameter values and printers’ settings that affect the microneedle topology, the authors were able to print a sharp microneedle with a tip radius of 15 μm. Another study of additive manufacturing was proposed by Krieger et al., which introduced a new method to overcome complications with such a technique [31]. The authors argued that in using the proposed method, it is possible to fabricate microneedles with high aspect ratios and sharp needle tips that are required to penetrate the skin. Wet and dry etching have also been used to fabricate microneedles. Ji et al. fabricated solid microneedles that are capable of penetrating the skin with less indentation [32].

The laser ablation method provides unique benefits as compared to other microneedle manufacturing methods. The laser ablation method uses a laser pulse as an energy source to remove a portion of the targeted solid parts [33,34]. The laser beam takes between 10 and 100 nanoseconds to fabricate the microneedle mold on the substrate [35]. This technique imparts desirable mechanical properties and high tensile strength of microneedle array [36]. Compared to photolithography and dry etching processes, laser ablation is a simple, rapid, low-cost laser processing method [37] with the ability to produce high-aspect-ratio microneedle master molds [38]. In utilizing a femtosecond laser, Evens et al. argued that the proposed method enables the production of low-cost microneedles due to their faster processing cycles and minimal laser maintenance, thereby reducing total life cycle costs over traditional methods [39]. Moreover, laser ablation provides significantly higher drug permeability in the receptor chamber and skin layers [40]. Thus, this paper investigated the laser ablation method for fabricating microneedles for transdermal drug delivery and therapeutic applications. Some of the limitations associated with the laser ablation method include the limited choice of fabricated materials [41]. Moreover, there is the possibility of producing contamination or toxicity during the interaction between the pulse and the targeted part [42].

Researchers have used different types of lasers to fabricate microneedle arrays. Tu and Chung used a CO_2_ laser to fabricate microneedle molds by performing two casting processes. Also, the excimer laser has been studied to investigate the capability of producing microstructure arrays [43]. Further, Zheng et al. used femtosecond laser pulses to study the effects of two temperature conditions on the hole’s geometry [44]. However, this paper proposed a new laser type for manufacturing microneedle molds (arrays) for transdermal drug delivery applications. An ytterbium laser was used to create the microneedle molds with polymethyl methacrylate (PMMA) material due to its higher melting efficiency performed with lower power values [45]. Compared to CO_2_, ytterbium offers an advantage that includes effective interaction with the target, less energy consumption, and high productivity [46]. Compared to other types of lasers, the ytterbium laser offers a high optical quality, a compact size, an extended lifetime, and a flexible mode of operation [47].

Aldawood et al. asserted that there was no investigation conducted to study optimal process parameters for the fabrication of microneedles [48]. This paper overcomes this gap by investigating the fabrication approaches of microneedle arrays based on the manipulation of five different process parameters. These include defining different sets of parameters, which are the laser power, pulse width, pulse repetition, pulse interval, and design profile (waveform). Also, this research provided a framework for the design and manufacture of microneedles for therapeutic applications. Furthermore, the study included a predictive model for fabricating desired microneedle arrays with a specific dimension.

## 2. Materials and Methods

The goal of this study was to design and fabricate polymer microneedle molds using a laser ablation technique for the rapid casting of soft or dissolvable microneedles. The provided methods optimize microneedle geometries, test the mechanical properties of fabricated microneedles, and test the skin-piercing forces of polymer microneedles. This approach allows for the assessment of the potential of polymers for transdermal delivery and, further, their potential in the development and manufacture of dissolvable microneedles containing therapeutic peptides.

### 2.1. Materials

A flat sheet of polymethyl methacrylate (PMMA) material (McMaster-Carr, Aurora, OH, USA, Clear Scratch- and UV-Resistant Cast Acrylic Sheet SKU—8560K919) was used to develop the molds for the microneedle arrays. Due to its non-toxicity, low cost, easy processability, compatibility, and greater fracture resistance, PMMA is a promising polymer in biomedical applications and devices [49,50,51]. PMMA has also been approved by the FDA in medical applications [52]. Microneedles were cast using polydimethylsiloxane (PDMS), which has unique features such as favorable thermal stability and low thermal conductivity [53]. PDMS is also safe, biologically and chemically compatible, flexible, and offers adjustable stiffness and surface adhesion energy [54]. The PDMS (Dow SYLGARD™, Germantown, WI, USA, 184 Silicone Encapsulant Clear, Part number: 184 SIL ELAST KIT 0.5 KG, Thermal Conductivity: 0.16 W/mK, Viscosity: 3500) used in this study was prepared by mixing the polymer resin and a curing agent (weight ratio of 10:1) obtained from Ellsworth Adhesives.

### 2.2. Research Design

**Figure 1:**
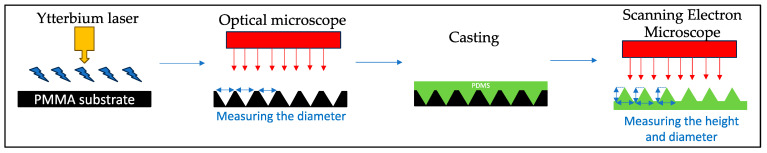
Schematic of the microneedle manufacturing process.

The ablation experiments were performed following the steps shown in Figure 1 using an ytterbium laser system (TLR-1070, IPG Photonics). The laser was focused on a flat sheet of PMMA substrate to create the mold by setting different parameters. IPG Photonics Pulse Shaper GUI 1.1 software was used to set the parameter values, and JR-C-Points software (version 2) was used to program the robot motion to guide the laser movement. The ablation relied on multiple variables, such as the laser power, the number of pulse repetitions, the interval between each pulse, the width of the pulse, and waveform type. A multi-axis Nordson EFD robot was used to guide the laser’s motion. A scanning electron microscope (SEM) was used to measure the diameter and height of the fabricated microneedle arrays. Once the PMMA substrate was ablated with the laser to form a microneedle master mold, it was followed by PDMS casting and releasing to obtain a flexible microneedle array. After that, the cast microneedle array was placed in an oven overnight at 45 °C to create an inverse mold. Sputter-coated needles were imaged using an SEM (Hitachi S-4800) where the shape, diameter, and height of the microneedles were acquired. These procedures were repeated to produce different microneedles based on different parameter values. The time it took to prepare a microneedle mold was less than 1 min using the laser, and it took around 24 h to prepare the microneedle array (casting).

#### 2.2.1. Pilot Study

A pilot study was designed to assess the quality of fabricated microneedle arrays. The input process parameters included waveform (square, triangle, or trapezoid shape), power (ranging from 0 to 200 watts), pulse width (ranging from 0 to 10 ms), repetitions (ranging from 0 to 500 times), and interval time (ranging from 0 to 200 ms). Due to the large range of parameter values, more than 200 experiments were conducted with different parameter values to optimize the data range. The height and diameter of microneedles were evaluated to validate the parameter values chosen. The prior literature has shown that a majority of microneedles have been fabricated with height values ranging between 0.5 and 3 mm and diameter values ranging between 0.1 and 0.25 mm [2,55]. These dimensions have been well suited for therapeutic applications for the penetration of the skin layers to deliver drugs. Thus, the microneedle dimension ranges chosen in our research are based on these findings in the literature. Also, the needle shape, surface roughness, and tip radius of microneedles were considered in the subsequent research.

#### 2.2.2. Design of Experiment

Based on the results of the pilot study, a design of experiment (DOE) was conducted to study the effects of the parameters on the outcomes. This experiment allowed for the evaluation of the factors and all possible combinations of factors on microneedle output quality. In this study, the DOE was defined with five input variables, each with two levels and three replicates (n = 3), for a total of 96 runs. Table 1 shows the input parameters and their levels. The outputs of this experiment were microneedle diameters and heights, which were captured using an SEM.

#### 2.2.3. Predictive Model

Based on the results from the experimental design, a regression analysis test was conducted to predict microneedle diameters and heights. Two regression equations were investigated: one for microneedle diameters and one for microneedle heights. The values of the input factors were entered as shown in Table 1, except the waveform values were replaced with 1 and 2 for the square and trapezoid, respectively. For the output, the values of diameter and height were captured from the Results Section 3. The predictive model using a linear equation followed the formula y = b_0_ + b_1_x_1_ + b_2_x_2_ + … + b_5_x_5_, where

- y denotes the predicted response for the experiments (diameter and height).
- b_0_ denotes the intercept coefficient.
- b_1_–b_5_ denote the coefficients of the respective input factors waveform, power, pulse width, repetition, and interval, respectively.
- x_1_–x_5_ denote the predictor values for the respective experiment input factors: waveform, power, pulse width, repetition, and interval, respectively.

#### 2.2.4. Mechanical Characterization

The microneedles fabricated using laser ablation were characterized using a mechanical compression test to simulate penetration within the skin. The microneedles were subjected to an axial force in a universal testing machine (Instron^®^ Model 5542, Instron, Norwood, MA, USA) to study the relation between the load applied to the displacement in compression mode. The Instron test station has a ±0.1% crosshead speed accuracy, ±0.015 mm position repeatability, and ±0.4% load measurement accuracy. A compression force was applied parallel to the microneedle height (*y*-axis) with a speed of 0.5 mm/s. The needle was placed on the bottom fixture of the station. The bottom of each single needle was glued onto the circular glass base and placed on the bottom fixture of the station. The outcome of this test was to identify the values of the force versus the displacement of the microneedle.

## 3. Results and Discussions

Microneedle arrays were fabricated using an ytterbium laser in polymers, followed by a pilot study showcasing design parameters, mechanical testing outcomes, predictive experimental modeling, and statistical testing. Certain sets of parameters were identified to have better microneedle experimental outcomes with desired geometries. The PDMS needles were primarily used to understand the ablation outcomes to benefit predictive modeling and analysis. Moreover, SEM was used to capture the microneedles’ surface, diameter, and height. Finally, several statistical treatments, including a normality check, response surface, and regression analysis were conducted to draw a conclusion about the capabilities of printed microneedle arrays.

### 3.1. Pilot Study

After conducting a pilot study, we were able to validate the optimal parameters and their values for further investigation. The results of the pilot study are summarized in Table 2 and explained in subsequent paragraphs.

#### 3.1.1. Waveform

**Figure 2:**
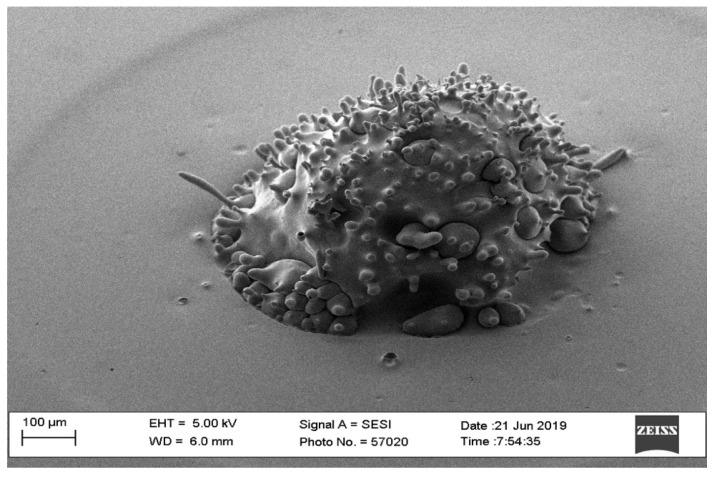
SEM image of a failed microneedle with parameters of triangle waveform and 40-watt laser power.

**Figure 3:**
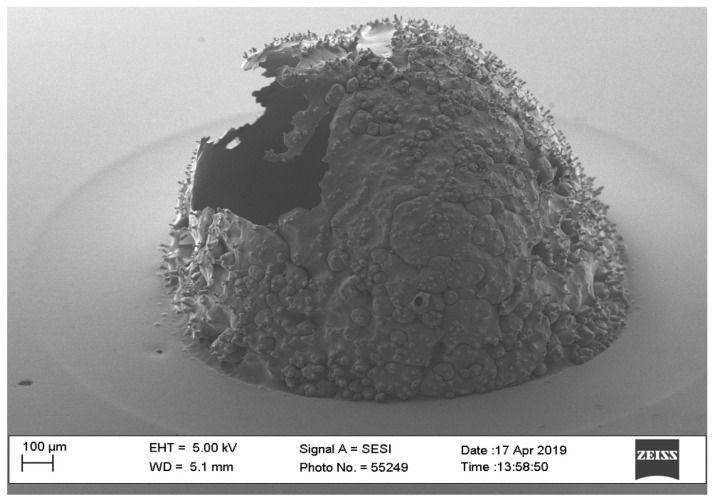
SEM image of microneedle with parameters of triangle waveform and 60-watt laser power.

The optimal way of fabricating microneedles in the shape of a pyramid or cone was to use a trapezoid waveform. The option of a square waveform resulted in an optimally shaped microneedle. However, the triangle waveform was not considered an optimal choice since it requires a high-powered beam, resulting in a larger diameter (>2 mm). The findings of the pilot study showed that the triangle waveform required at least 40 watts of laser power to produce a needle; however, it needed more power to produce a pyramid or cone shape. Figure 2 shows a needle that has a triangle waveform with a 40-watt laser power, a 2 mm pulse width, 100 repetitions, and a 0 mm interval time. The microneedle shows a hole due to casting and, thus, this microneedle was excluded from our scope based on the preliminary pilot study. On the other hand, when the power is 60 watts, the triangle form shows a good outcome for the needles (Figure 3).

#### 3.1.2. Laser Power

**Figure 4:**
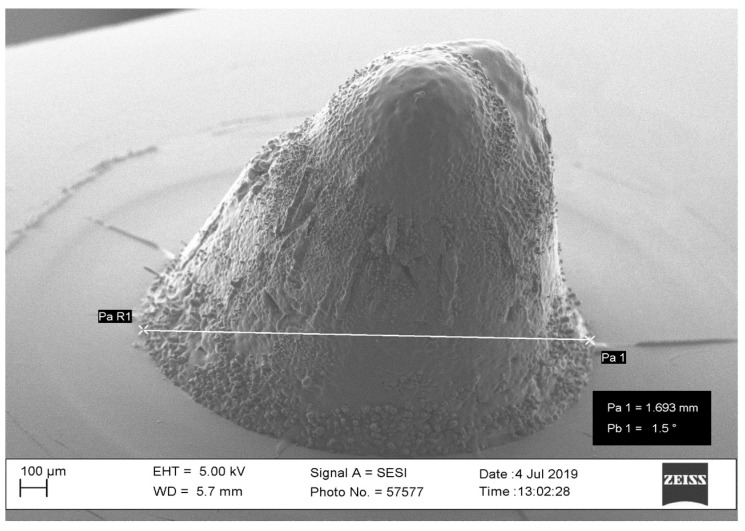
SEM image of microneedle fabricated with 60-watt laser power.

The laser power value should be in the range of 30 to 40 watts to obtain the desired outcomes for microneedles. Microneedle dimensions should range between 0.5 and 3 mm in height and 0.1 and 0.25 mm in width to be effective in therapeutics [56,57]. Power below 30 watts will not result in any holes and is considered too low to create a needle. Power above 40 watts will result in a large diameter (out-of-specification). Figure 4 shows a needle with a diameter of 1.69 mm created with 60-watt laser power. The laser power is to be used in conjunction with the laser waveform being implemented during microneedle fabrication. From our pilot study, it was revealed that a triangle waveform produced inconsistent microneedles and resulted in a shorter aspect ratio (height to diameter ratio). The trapezoidal and square waveforms that were considered for the final design of experiments were conducted at 30 W and 40 W powers. As higher powers beyond 40 W resulted in larger microneedles (beyond 2 mm in height) these are not effective for therapeutics.

#### 3.1.3. Pulse Width

**Figure 5:**
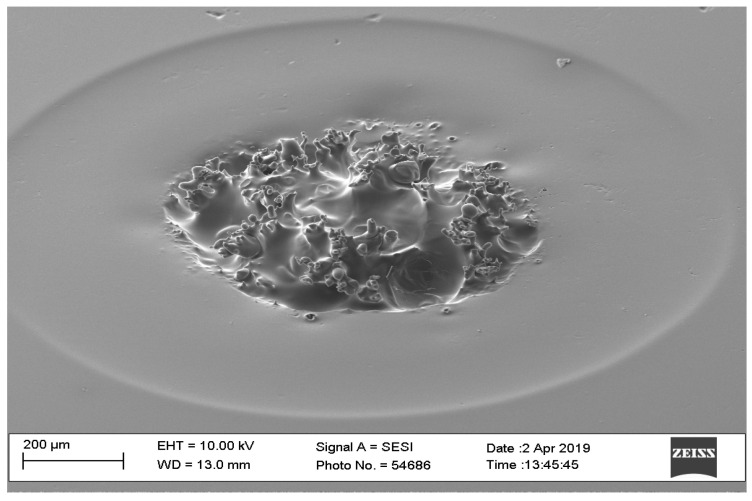
SEM image of failed microneedle fabricated with 1.5 ms pulse width and 80-watt laser power.

The optimal range for pulse width was from 3 to 5 ms to approach the desired outcome. Higher values of pulse width resulted in a larger needle diameter. A successful microneedle with a 1 mm diameter was fabricated with a 10 ms pulse width. However, reducing pulse widths to 1.5 ms with higher laser power (80 watts) resulted in poor outcomes due to an extremely short time duration of excitation, as shown in Figure 5.

#### 3.1.4. Number of Repetitions

The optimal values of repetitions ranged between 50 and 100 times. If the number of repetitions was more than 100, it resulted in a large needle diameter. Variations in the number of repetitions with the constant set of the other parameters (square shape form, 30-watt laser power, 5 ms pulse width, and 0 ms interval) resulted in a microneedle diameter of 0.23 mm with 20 repetitions, 0.55 mm with 50 repetitions, 0.65 mm with 100 repetitions, and 0.77 mm with 150 repetitions, respectively.

#### 3.1.5. Interval Time

**Figure 6:**
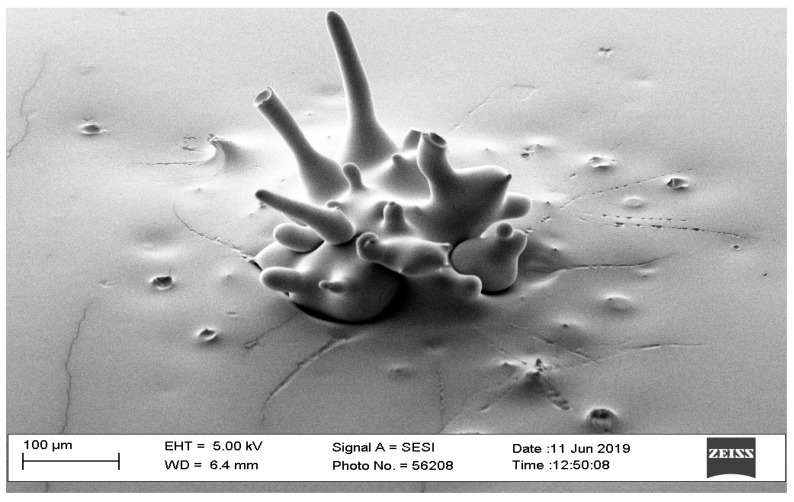
SEM image of a failed microneedle with an interval time of 100 ms.

The optimal values of laser interval time were from 0 ms to 50 ms. A higher interval time will not be able to create microneedle arrays. For this part of the pilot study, the first finding on the optimal interval time concluded that the diameters of the needles were 0.85 mm and 0.3 mm for 0 ms and 50 ms interval times, respectively. Figure 6 shows that an interval time of 100 ms did not result in a microneedle.

### 3.2. Design of Experiments

Based on the results of the pilot study, two values of each parameter were chosen for further study and investigation. These include waveform (square and trapezoid), power (30 and 40 watts), pulse width (3 and 5 ms), repetition (50 and 100 times), and interval (0 and 50 ms). Using these parameter values, three runs (n = 3) of 32 microneedles were fabricated for further study. The results of the microneedle designs (Table 3) show that all parameter sets produced well-designed microneedles except microneedle number 18. Microneedle number 18 had a trapezoidal shape, 30-watt power, 3 mm pulse width, 50 repetitions, and 50 mm interval time. This microneedle confirms our hypothesis that microneedles are not formed when the process parameters are below their lowest threshold. In this case, the increase in the interval of lasing to 50 ms resulted in lower energy levels and, thereby, not producing a microneedle structure.

**Figure 7:**
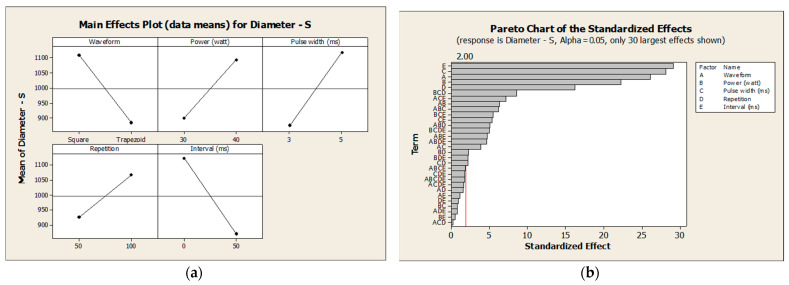
(**a**) Main effects plot for diameter and (**b**) Pareto chart of the standardized effects on the diameter.

**Figure 8:**
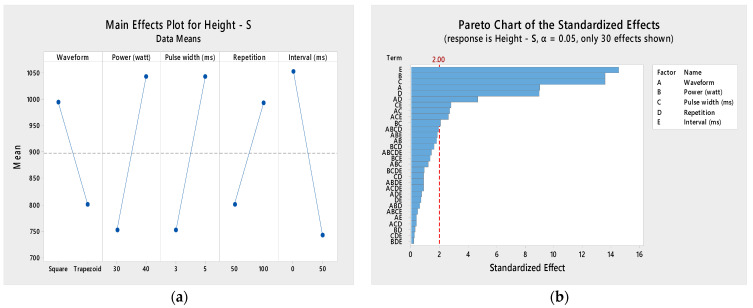
(**a**) Main effects plot for height and (**b**) Pareto chart of the standardized effects on the height.

Figure 7a and Figure 8a show that the square waveform has the highest impact on the diameter and height compared to the trapezoid shape. Figure 7b and Figure 8b show that all input factors were statistically significant at the 0.05 level with the current model. Some interactions between factors have a significant impact on the diameter and height of the needles. Increasing the values for laser power and pulse width will result in increasing the microneedle’s diameter and height. Increasing the time that a microneedle array is exposed to the laser will result in increasing the microneedle’s diameter and height. Finally, the interval between laser pulses has an inverse relationship to the microneedle’s diameter and height.

Table 4 shows that the interval factor has the highest effect (252.229) on the diameter of the microneedle, with an inverse relationship. The second highest factor that impacts the diameter is the pulse width (243.521). Table 5 shows that the interval has the highest effect (310.833) on the height of microneedles, with an inverse relationship. The power and pulse width accounted for significant factors in the experiment. The waveform affects both the diameter and the height of microneedles. However, this factor has a lower impact on the outcomes compared to other factors. Figure 8 shows that the square waveform is associated with the highest mean diameter and height. On the other hand, the trapezoid waveform is associated with a lower mean for both the diameter and the height of microneedles.

The laser power factor is also a significant factor in this experiment. The laser power has the highest effect on the height of the microneedles but a lower effect on the diameter. Increasing the value of the laser power will create a taller needle (direct correlation). Changing the value of the laser pulse width is a significant factor in this experiment. This factor is considered the second-highest significant factor in the outcomes. The pulse width affects the height slightly more than it affects the diameter. We can conclude that the diameter and height of microneedle designs have a direct correlation with the pulse width factor. Increasing the pulse repetition will result in increasing the diameter and height of the microneedles. However, this factor has the lowest effect values on the outcomes compared to other factors. The pulse interval is a significant factor in this experiment. The pulse interval factor has the highest effect in the experiment on the diameter and the height. The interval factor has an inverse relationship with the values for diameter and height—50 ms was associated with the lowest mean of diameter and height and 0 ms was associated with the highest mean.

There were 14 interactions between factors considered to have a significant effect on the diameter of microneedles (Figure 8). On the other hand, only seven interactions between factors were considered to have a significant impact on height. These interactions had lower impacts on the outcomes of the experiment. The interactions between factors were seen to affect the diameter more than the height of microneedles.

#### 3.2.1. Normality Check

**Figure 9:**
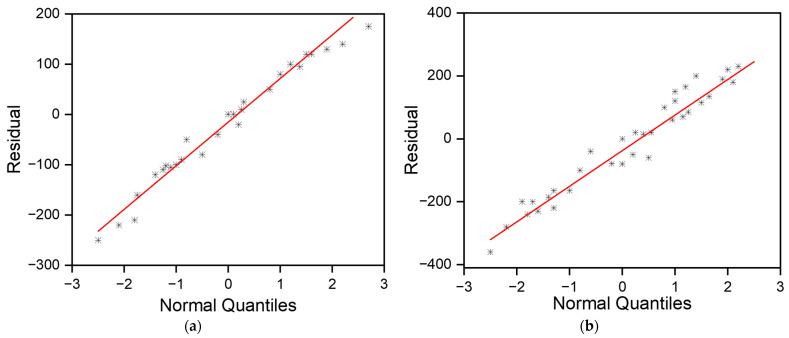
Normality plot for microneedle (**a**) diameter and (**b**) height.

**Figure 10:**
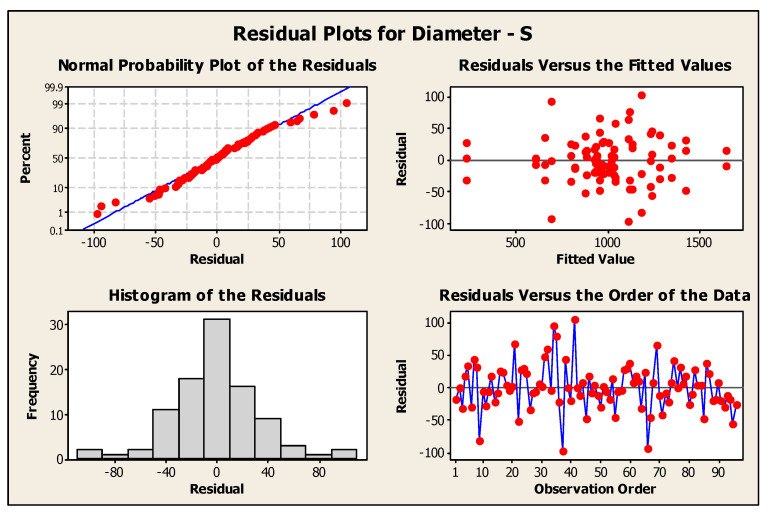
Residual plots for microneedle diameter.

**Figure 11:**
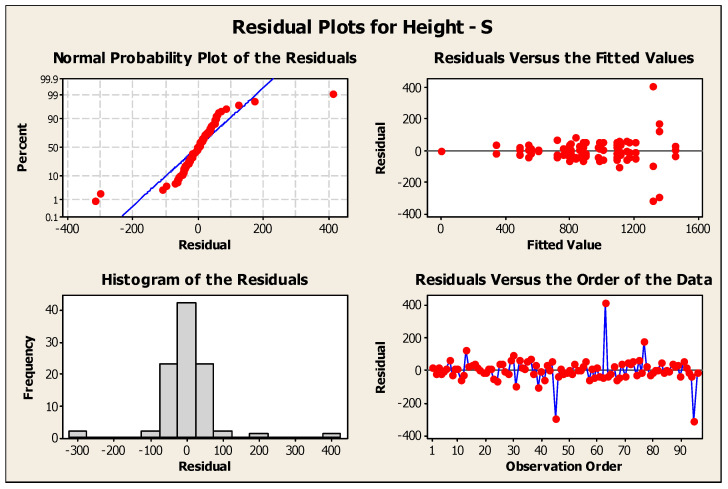
Residual plots for microneedle height.

The null hypothesis (H_0_) for any normality test is that the data are normally distributed or from a Gaussian distribution. If the *p*-value is <0.05, then it indicates that the null hypothesis is rejected in favor of the alternate hypothesis (H_1_), which states that the data are not normally distributed. The Shapiro–Wilk test for both the diameter and height datasets had *p*-values of 0.1746 and 0.13, respectively. Thus, the null hypothesis failed to be rejected, which indicated that all variables were normally distributed. Moreover, the *p*-values of the Kolmogorov–Smirnov test were 0.15 and 0.281 for the diameter and height, respectively. This also concluded that the data were normally distributed. Figure 9 shows that the experiment was well modeled using a normal distribution, and the random variables underlying the dataset were normally distributed. Figure 10 and Figure 11 show residual plots for both diameter and height, respectively, which indicate that the data are not skewed, have nonconstant variance, and are independent. Both the histograms are symmetrical, showing a better-fit regression model, and the random order of residuals with fitted and observation orders indicates no correlation among themselves. 

#### 3.2.2. Response Surface Analysis

**Figure 12:**
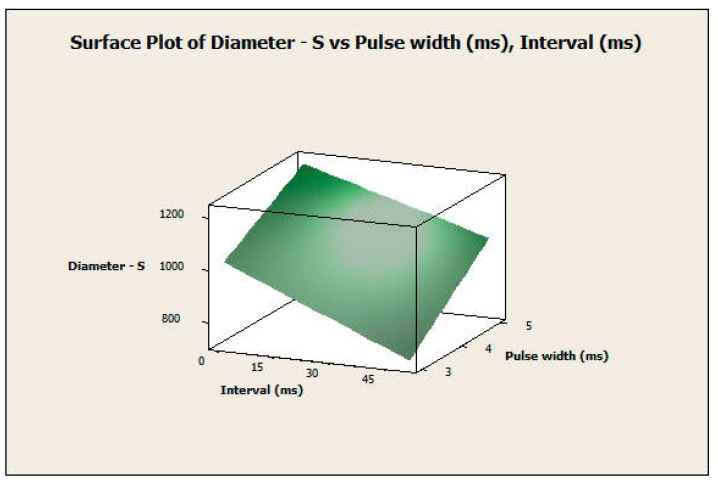
Surface plot of diameter versus pulse width and interval.

**Figure 13:**
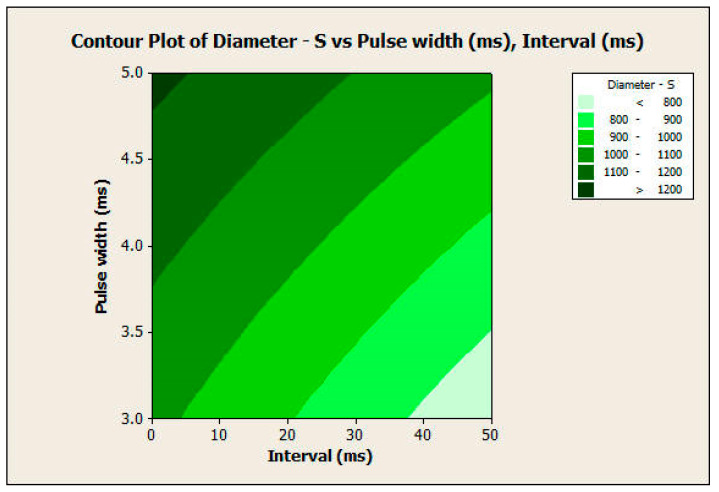
Contour plot of diameter versus pulse width and interval.

The response surface analysis was conducted for both the diameter and height of the microneedles using a central composite design. The two most significant factors in both experiments, which include pulse width and interval, were considered input variables. Figure 12 and Figure 13 show the surface and contour plots for the diameter. The highest values of the diameter for the microneedle array were in the top-left corner of the plot (Figure 13), which corresponds with a high value of pulse width and a low value of interval. The lowest values of the diameter for the microneedle array were in the lower-right corner of the plot, which corresponds with low values of pulse width and high values of interval.

**Figure 14:**
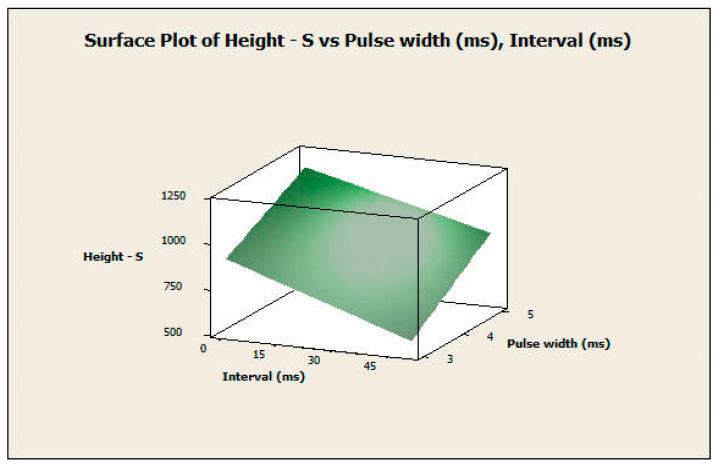
Surface plot of height versus pulse width and interval.

**Figure 15:**
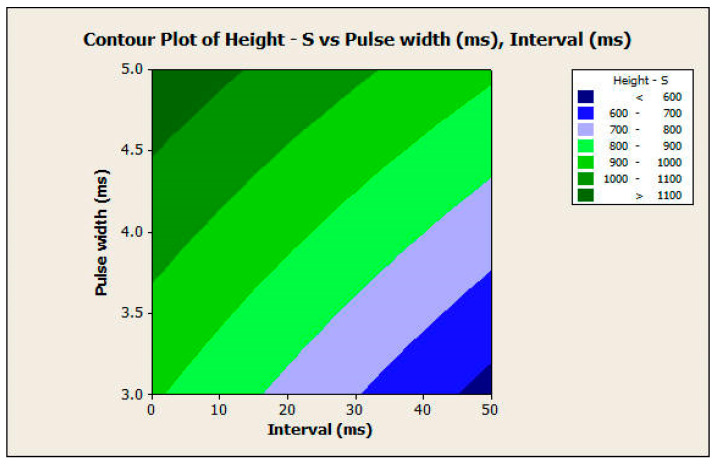
Contour plot of height versus pulse width and interval.

This finding also applies to the response surface analysis of the height of the microneedle array in the same manner (Figure 14 and Figure 15). In this analysis, the null hypothesis (H_0_) states that there is no relationship between the independent variables (pulse width and pulse interval) and the dependent variable (response variable). The *p*-values of the pulse width, interval, and the interaction between them were 0.000 for microneedle diameter. The *p*-values of the pulse width, interval terms, and interaction between them were 0.000, 0.000, and 0.007, respectively, for microneedle height. Since the *p*-values are less than 0.05, we reject the null hypothesis in favor of the alternate hypothesis, which states that pulse width, pulse interval, and their interactions have a significant influence on the response variables of microneedle diameter and height. Therefore, the full quadratic model of the pulse width and the interval factors (independent variables) significantly affect the response diameter and height (dependent variable). Thus, one can obtain specific values of microneedle diameter and height by choosing a combination of input process parameters.

#### 3.2.3. Predictive Model

A regression model was conducted to predict the relationship between the independent variables and the dependent variables. The ANOVA test with a 95% confidence level shows that the *p*-value is less than 0.05, which indicates that it is statistically significant (Table 6 and Table 7). Table 8 and Table 9 show the regression statistics and ANOVA for the input of the microneedle diameter. Table 10 and Table 11 show the input data of regression statistics and ANOVA for the microneedle height. From both Table 8 and Table 10, R values were more than 0.9, which indicated that the variance of the parameters was studied and reflected well on the variance of the output. Equation (1) represents a regression model for the diameter of the microneedles, and Equation (2) represents the predictive model for the height of the microneedles. The predictive model equations can give tentative values of diameter and height for a set of chosen input parameters. Moreover, the F-value and *p*-value of the regression models in Table 9 and Table 11 indicated that both models could be represented on larger population data. The parameter coefficients in Equations (1) and (2) show a generalized model encompassing the limits of feasible process parameter values for the five process parameters (laser waveform, laser power, pulse width, pulse repetition, and interval between pulses). Any microneedles beyond the ranges chosen in this research would result in a “failed” microneedle as discussed earlier. Thus, these equations provide a direct relationship between the parameters and the outputs (diameter and height). These parametric equations serve as a guide for both novice designers and seasoned pharmaceutical professionals to choose process parameters that suit their application.

Diameter (μm) = 89.14 − 226.60 × Waveform + 19.32 × Power + 121.76 × Pulse width + 2.80 × Repetition − 5.04 × Interval(1)

Height (μm) = −544.87 − 193.25 × Waveform + 29.07 × Power + 145.31 × Pulse Width + 3.84 × Repetition − 6.21 × Interval(2)

Finally, a post hoc analysis was conducted using Tukey’s Studentized Range (HSD) Test (Table 12). The findings of this test emphasized the ANOVA test results, where the means are having a significant impact on the fabricated microneedle.

### 3.3. Mechanical Characterization of Microneedles

**Figure 16:**
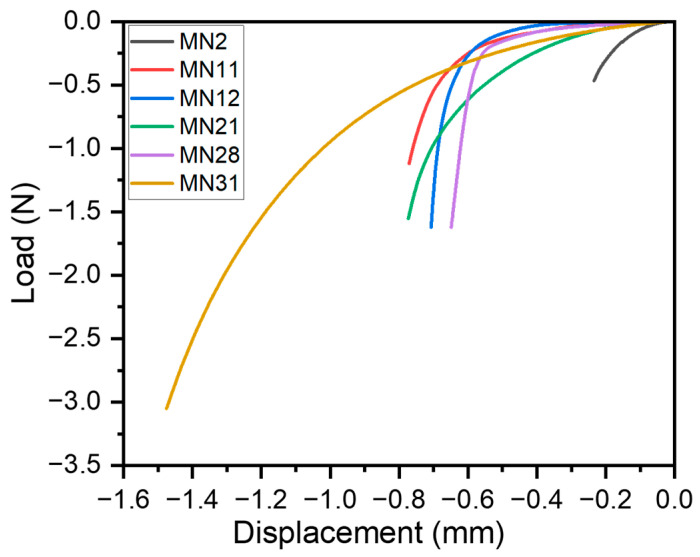
Demonstration of microneedle displacement for selected microneedles.

The microneedles fabricated using a combination of different process parameters were investigated for mechanical deformation to simulate skin penetration. All microneedle designs shown in Table 3 were subjected to compressive loads along the height of the microneedle. Figure 16 shows six candidate microneedles based on distinct process parameters to show variation in their load–displacement curves. The microneedle number MN corresponds to the respective process parameters in Table 3.

MN2 corresponds to microneedle #2 (waveform: square, power: 30 W, pulse width: 3 ms, repetitions: 50, and interval: 50 ms). Other microneedles—MN11 through MN31—follow similar nomenclatures, and their process parameters can be observed in Table 3. MN2’s process conditions resulted in a microneedle with an average diameter and height of around 700 μm and 500 μm, respectively. Due to a short aspect ratio (AR)—which is the height-to-diameter ratio—of 0.71, this microneedle had a lower deformation of 0.23 mm at a maximum load of 0.47 N as seen in Figure 16. The relatively higher diameter as compared to the microneedle height resulted in a stiffer mechanical response and, thereby, the minimal deformation of the microneedle MN2. This can be attributed to its square laser waveform, lower power (30 W), pulse width (3 ms), lower laser repetition rate (50), and higher lasing interval (50 ms). In contrast, MN31 had the tallest height of around 1.5 mm and diameter of 1.25 mm due to its trapezoidal laser waveform, higher power (40 W), pulse width (5 ms), laser repetition rate (100), and shortest lasing interval (0 ms). This resulted in the highest deformation (1.48 mm) at a load of 3.05 N for MN31, for its aspect ratio of 1.25. Thus, high-aspect-ratio microneedles had higher deformation, which would correspond to higher penetration depths within the skin. A comparative analysis between MN11 and MN12 shows that an increase in the lasing interval between laser pulses results in shorter and smaller-diameter MNs. Thus, MN12, which had a smaller AR (AR = 0.86), required a higher deformation force of 1.66 N as compared to MN11 (AR = 0.91), with a deformation force of 1.12 N, respectively.

In general, process parameters following these trends had higher diameters and heights. These include trapezoidal waveform, higher laser power, longer pulse width, higher laser repetitions, and shorter lasing intervals. The microneedle design is based on the location and skin type of the human body as the penetration force varies on the stratum corneum. Longer and larger-diameter needles (MN 11 and MN 31) display a better ability to penetrate robust skin surfaces similar to the soles of feet, whereas shorter needles (MN2 and MN28) can penetrate delicate skin areas. As seen in Figure 16, all microneedle designs were able to satisfy the function of penetration at varying depths. Shah et al. have demonstrated using a solvent-casting process that the minimum force for a 100-microneedle array was around 4 N [58]. Ning et al. reported that the minimum force required to puncture the human skin with a single microneedle was around 5.8 N [59]. Hyaluronic acid (HA) was used by Chi et al. to develop different dissolvable microneedles. Based on the molecular weight of the HA, they obtained a load of 21.6, 13.9, and 5.9 N per array of 100 microneedles, for 10 k-HA- MN, 74 k-HA-MN, and 290 k-HA-MN, respectively [60]. Based on our findings, researchers and practitioners can, thereby, design custom-aspect-ratio needles by varying multiple process parameters as shown in Table 3 to obtain relevant mechanical deformation based on their application intent in the therapeutic setting.

### 3.4. Discussion and Future Directions

In this study, we propose a new type of laser ablation method using the ytterbium laser. Many studies have used CO_2_ as the type of laser to fabricate microneedles; however, Osipov et al. argued that the ytterbium laser is more efficient compared to CO_2_ laser [61]. Moreover, the ytterbium laser consumed less energy and caused less change in the composition of the nanoparticle elements compared to the CO_2_ laser [62]. This study also investigated the effects of several different parameters on microneedle manufacturing. Even though many studies have used different parameters [38,63], these studies were limited to two or three parameters while our study explored a comprehensive relationship between five parameters and their effects. The primary aim of this research was to investigate process parameters for a new laser type (ytterbium) for the fabrication of precision microneedles. Ytterbium lasers offer ultra-high-speed processing speeds (femtoseconds) and flexible on-the-fly parameter changes and provide a greener manufacturing practice due to their higher efficiencies [64,65]. Based on our literature search, this is the first time that this laser type has been implemented for microneedle fabrication and, thus, we explore its utility. Our research provides guidance to both researchers and practitioners on how to implement this laser for the customized design and fabrication of microneedles to suit their application intent.

**Figure 17:**
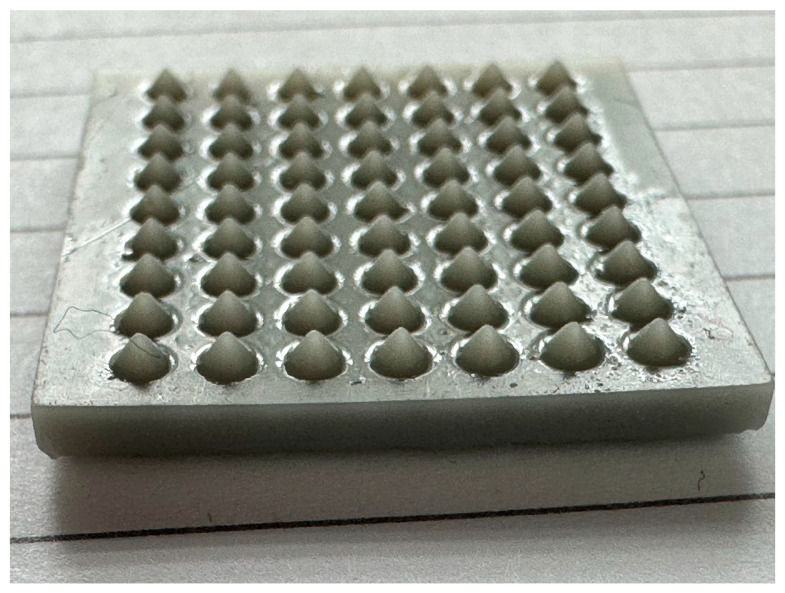
Microneedle array fabricated using ytterbium laser.

To overcome the limitations associated with the lack of literature and clear guidance in the matter of fabricating microneedle arrays, the study generated predictive regression-based models to generate a microneedle with the desired geometric profile. This predictive model will provide insight for practitioners and researchers to custom design microneedles using the ytterbium laser ablation method. Figure 17 shows a full patch (7 × 9 = 63 microneedles) fabricated using the ytterbium laser. As can be seen in the figure, a consistent and smooth microneedle patch was developed with a suitable aspect ratio. The process conditions to fabricate this microneedle patch include the following: laser waveform (square), power (40 W), pulse width (5 ms), repetition (100), and interval (0 ms). The future directions of this study include drug loading, drug release, and in vitro microneedle degradation studies.

## 4. Conclusions

A new approach to fabricating microneedles using an ytterbium laser was proposed in this research. A pilot study was conducted to optimize the parameters’ values in order to run a design of experiments by observing the dimensions, profile surface finish, and quality of the needles. This study also investigated the effects of different parameters on the diameter and height of the needles by conducting several statistical tests. Different tests were conducted to draw conclusions from this research, such as response surface design and a full design of experiments. The outcomes of this research showed that all parameters had a significant effect on the experiment. The laser power, pulse width, and the number of repetitions have correlations with the outcomes (diameter and height of the microneedle). In contrast, the interval time between laser beams has an inverse relationship with diameter and height. The most significant factors in the experiment were the laser pulse width and interval time. The trapezoid waveform has a lower impact on the outcomes compared to the square waveform. A predictive model for fabricating the desired geometry of diameter and height of a microneedle array was developed. This research provides a procedural framework for designing and manufacturing microneedle arrays for transdermal drug delivery applications.

## Figures and Tables

**Table 1:** Input parameters and their levels.

Parameters	Level 1	Level 2
Waveform	Square	Trapezoid
Laser power	30 w	40 w
Laser pulse width	3 ms	5 ms
Number of repetitions	50 times	100 times
Interval time	0 ms	50 ms

**Table 2:** Pilot study summary.

Parameter	Values Available	Optimal Choice	Reason	Values to Exclude
**Waveform**	Square, triangle, or trapezoid shape	Trapezoid or Square	A trapezoid will result in having a pyramid or cone shape. A square will result in a fair needle shape.	Triangle form required high laser power values, which resulted in a large diameter of the needle.
**Power (watts)**	0 watts to 200 watts	30 watts to 40 watts	Seek desired outcomes	Power below 30 watts will not result in any holes and is too low to create a needle. Higher power will cause a larger diameter.
**Pulse Width (ms)**	0 to 10 ms	3 to 5 ms	Seek desired outcomes	Values higher than 5 ms will cause a larger diameter.
**Number of repetitions**	0 to 500 times	50 to 100 times	Seek desired outcomes	Values higher than 100 times will cause a larger diameter.
**Interval time (ms)**	0 to 200 ms	0 to 50 ms	Seek desired outcomes	Values higher than 50 ms will cause a larger diameter.

**Table 3:** Microneedle diameter and height results.

	Parameters					Run 1		Run 2		Run 3	
No.	Waveform	Power (watts)	Pulse Width (ms)	Repetitions	Interval (ms)	Diameter (μm)	Height (μm)	Diameter (μm)	Height (μm)	Diameter (μm)	Height (μm)
1	Square	30	3	50	0	917	800	931	796	958	762
2		30	3	50	50	687	456	782	492	593	505
3		30	3	100	0	1083	995	1193	1033	1068	917
4		30	3	100	50	897	691	858	788	887	673
5		30	5	50	0	1144	1105	1013	1089	1175	1153
6		30	5	50	50	922	871	997	893	940	821
7		30	5	100	0	1278	1171	1235	1002	1193	1155
8		30	5	100	50	1004	856	952	876	964	924
9		40	3	50	0	1100	1011	1287	945	1161	1057
10		40	3	50	50	933	808	939	829	948	766
11		40	3	100	0	1250	1094	1267	1154	1319	1213
12		40	3	100	50	1013	860	1027	949	1019	877
13		40	5	50	0	1441	1481	1377	1057	1456	1532
14		40	5	50	50	1009	997	1049	933	1038	998
15		40	5	100	0	1638	1485	1638	1461	1663	1422
16		40	5	100	50	1372	1157	1350	1095	1320	1103
17	Trapezoid	30	3	50	0	843	577	806	545	808	560
18		30	3	50	50	230	0	195	0	254	0
19		30	3	100	0	928	785	934	779	937	844
20		30	3	100	50	605	323	597	374	607	323
21		30	5	50	0	1018	810	932	800	903	803
22		30	5	50	50	827	610	892	605	916	598
23		30	5	100	0	1157	1035	1084	1108	1151	1125
24		30	5	100	50	1000	815	964	934	950	902
25		40	3	50	0	953	835	927	735	913	824
26		40	3	50	50	760	575	820	543	801	496
27		40	3	100	0	992	1206	1029	1165	981	1267
28		40	3	100	50	649	728	692	770	624	770
29		40	5	50	0	1039	1148	1040	1051	1022	1076
30		40	5	50	50	930	924	946	788	909	797
31		40	5	100	0	1286	1223	1248	1734	1184	1005
32		40	5	100	50	1101	1224	1009	1126	1015	1154

**Table 4:** Parameter Effect Values on Microneedle Diameter.

Observation	Effect	est1	Effects
1	A	−113.30	−226.60
2	B	96.61	193.22
3	C	121.76	243.52
4	D	70.11	140.22
5	E	−126.11	−252.22
6	AB	−27.19	−54.39
7	AC	16.53	33.06
8	AD	−6.78	−13.56
9	AE	4.82	9.64
10	BC	−3.51	−7.02
11	BD	−9.94	−19.89
12	BE	2.40	4.81
13	CD	9.49	18.97
14	CE	23.21	46.43
15	DE	−4.30	−8.60
16	ABC	−26.94	−53.89
17	ACD	1.13	2.27
18	ABD	−22.13	−44.27
19	ABE	20.63	41.27
20	ACE	31.19	62.39
21	ADE	−3.49	−6.97
22	BCD	37.34	74.68
23	BCE	−23.84	−47.68
24	BDE	−9.65	−19.31
25	CDE	−7.55	−15.10
26	ABCD	0.53	1.06
27	ABCE	−8.07	−16.14
28	ABDE	−20.13	−40.27
29	ACDE	−7.03	−14.06
30	BCDE	21.67	43.35
31	ABCDE	7.40	14.81

**Table 5:** Parameter Effect Values on Microneedle Height.

Observation	Effect	est1	Effects
1	A	−96.62	−193.25
2	B	145.37	290.75
3	C	145.31	290.62
4	D	96.10	192.20
5	E	−155.41	−310.83
6	AB	19.39	38.79
7	AC	29.08	58.16
8	AD	50.12	100.25
9	AE	−4.18	−8.37
10	BC	−22.25	−44.50
11	BD	−3.41	−6.83
12	BE	−0.85	−1.70
13	CD	−9.60	−19.20
14	CE	29.79	59.58
15	DE	7.79	15.58
16	ABC	−13.14	−26.29
17	ACD	4.00	8.00
18	ABD	6.35	12.70
19	ABE	19.87	39.75
20	ACE	28.10	56.20
21	ADE	−7.89	−15.79
22	BCD	17.20	34.41
23	BCE	−14.31	−28.62
24	BDE	−2.18	−4.37
25	CDE	2.83	5.66
26	ABCD	−20.60	−41.20
27	ABCE	−5.00	−10.00
28	ABDE	−9.45	−18.91
29	ACDE	9.39	18.79
30	BCDE	9.77	19.54
31	ABCDE	15.41	30.83

**Table 6:** ANOVA Results for Diameter.

	Coefficients	Standard Error	t Stat	*p*-Value	Lower 95%	Upper 95%	Lower 95.0%	Upper 95.0%
Intercept	89.13	91.36	0.97	0.33	−92.36	270.63	−92.36	270.63
Waveform	−226.60	19.81	−11.43	0.00	−265.97	−187.23	−265.97	−187.23
Power	19.32	1.98	9.74	0.00	15.38	23.26	15.38	23.26
Pulse width	121.76	9.90	12.28	0.00	102.07	141.44	102.07	141.44
Repetition	2.80	0.39	7.07	0.00	2.01	3.59	2.01	3.59
Interval	−5.04	0.39	−12.72	0.00	−5.83	−4.25	−5.83	−4.25

**Table 7:** ANOVA Results for Height.

	Coefficients	Standard Error	t Stat	*p*-Value	Lower 95%	Upper 95%	Lower 95.0%	Upper 95.0%
Intercept	−544.87	121.12	−4.49	0.00	−785.50	−304.24	−785.50	−304.24
Waveform	−193.25	26.27	−7.35	0.00	−245.45	−141.04	−245.45	−141.04
Power	29.07	2.62	11.06	0.00	23.85	34.29	23.85	34.29
Pulse width	145.31	13.13	11.06	0.00	119.21	171.41	119.21	171.41
Repetition	3.84	0.52	7.31	0.00	2.80	4.88	2.80	4.88
Interval	−6.21	0.52	−11.83	0.00	−7.26	−5.17	−7.26	−5.17

**Table 8:** Effects Values for the Factors on Diameter.

**Multiple R**	0.931
**R Square**	0.867
**Adjusted R Square**	0.860
**Standard Error**	97.091
**Observations**	96

**Table 9:** ANOVA Contributions on Diameter.

	**df**	**SS**	**MS**	**F**	***p* Value**
**Regression**	31	6,283,437.91	202,691.55	112.28	<0.0001
**Residual**	64	115,533.33	1805.21		
**Total**	95	6,398,971.24			

**Table 10:** Effects Values for the Factors on Height.

**Multiple R**	0.919
**R Square**	0.845
**Adjusted R Square**	0.836
**Standard Error**	128.721
**Observations**	96

**Table 11:** ANOVA Contributions on Height.

	df	SS	MS	F	*p* Value
**Regression**	31	8,949,791.96	288,702.96	26.43	<0.0001
**Residual**	64	699,162.00	10,924.41		
**Total**	95	9,648,953.96			

**Table 12:** Tukey’s Studentized Range (HSD) test.

Tukey’s Studentized Range (HSD) Test for Output				
Alpha			0.05	
Error Degrees of Freedom			64	
Error Mean Square			10,924.41	
Critical Value of Studentized Range			2.82522	
Minimum Significant Difference			42.622	
**Means with the same letter are not significantly different.**				
Tukey Grouping	Mean	N	Waveform	
A	993.65	48	1	
B	800.40	48	2	
Tukey Grouping	Mean	N	Power	
A	1042.40	48	40	
B	751.65	48	30	
Tukey Grouping	Mean	N	Pulse	
A	1042.33	48	5	
B	751.71	48	3	
Tukey Grouping	Mean	N	Repetition	
A	993.13	48	100	
B	800.92	48	50	
Tukey Grouping	Mean	N	Interval	
A	800.40	48	0	
B	800.40	48	50	

## Data Availability

The data presented in this study are available upon request from the corresponding author. The data are not publicly available due to proprietary formulation.
